# The Effect of Ignoring Statistical Interactions in Regression Analyses Conducted in Epidemiologic Studies: An Example with Survival Analysis Using Cox Proportional Hazards Regression Model

**DOI:** 10.4172/2161-1165.1000216

**Published:** 2015-01-15

**Authors:** KP Vatcheva, M Lee, JB McCormick, MH Rahbar

**Affiliations:** 1 Division of Epidemiology, University of Texas Health Science Center-Houston, School of Public Health, Brownsville Campus, Brownsville, TX, USA; 2 Department of Epidemiology, Human Genetics, and Environmental Sciences (EHGES), University of Texas School of Public Health at Houston, Houston, TX, USA; 3 Division of Clinical and Translational Sciences, Department of Internal Medicine, Medical School; The University of Texas Health Science Center at Houston, Houston, TX, USA; 4 Biostatistics/Epidemiology/Research Design (BERD) Core, Center for Clinical and Translational Sciences (CCTS), The University of Texas Health Science Center at Houston, Houston, TX, USA

**Keywords:** Effect modification, Cox proportional hazards model, Regression analysis, Simulation, Statistical interaction, Type 2 diabetes

## Abstract

**Objective:**

To demonstrate the adverse impact of ignoring statistical interactions in regression models used in epidemiologic studies.

**Study design and setting:**

Based on different scenarios that involved known values for coefficient of the interaction term in Cox regression models we generated 1000 samples of size 600 each. The simulated samples and a real life data set from the Cameron County Hispanic Cohort were used to evaluate the effect of ignoring statistical interactions in these models.

**Results:**

Compared to correctly specified Cox regression models with interaction terms, misspecified models without interaction terms resulted in up to 8.95 fold bias in estimated regression coefficients. Whereas when data were generated from a perfect additive Cox proportional hazards regression model the inclusion of the interaction between the two covariates resulted in only 2% estimated bias in main effect regression coefficients estimates, but did not alter the main findings of no significant interactions.

**Conclusions:**

When the effects are synergic, the failure to account for an interaction effect could lead to bias and misinterpretation of the results, and in some instances to incorrect policy decisions. Best practices in regression analysis must include identification of interactions, including for analysis of data from epidemiologic studies.

## Introduction

The terms interaction effect and effect modification, or effect-measure modification are often used interchangeably, particularly for health related research in epidemiology [1]. From an epidemiologic prospective effect modification refers to a situation where the effect of one predictor variable (e.g., exposure) on the outcome is dependent on the values of some other covariates [1,2]. From a statistical prospective, an interactive multivariable regression model could be fit by including the product of two or more predictor variables along with their corresponding individual variables in regression models [1].

Identification of statistical interactions in a regression model is important because this could lead to significant public health implications. For example, using data from an Epidemiologic Study on the Insulin Resistance Syndrome (DESIR) cohort, Gautier A. et al. (2010) found significant interactions between baseline BMI categories and higher waist circumference in relation to progression to type 2 diabetes using a logistic regression model [3]. Based on their findings the authors concluded that for reducing incidence of type 2 diabetes in their study population, it is important to monitor and prevent increases in waist circumference, particularly for those with BMI <25 kg/m^2^. Similarly, Hanai K. et al. examined whether obesity modifies the association of serum leptin levels with the progression of diabetic kidney disease by including an interaction term between the obesity and leptin in a Cox proportional hazards regression model [4]. The authors detected a significant interaction between leptin levels and obesity, hence concluded that obesity modifies the effect of leptin on kidney function decline in patients with type 2 diabetes. On the other hand, in an Iranian sex-stratified cohort study which was conducted to investigate risk factors for incidence of type 2 diabetes using Cox proportional hazards regression model [5], the authors recommended different preventive strategies by sex because of their findings that along with central adiposity in women and general adiposity in men, a lower education level conferred a higher risk for incidence of diabetes among men. Using data from Second Manifestations of Arterial Disease (SMART) study, Verhagen S. N. et al. investigated the relation between high-sensitivity C-reactive protein (hsCRP) levels and the incidence of type 2 diabetes in patients with manifest arterial disease or any cardiovascular risk factors [6]. The authors identified a significant interaction between gender and hsCRP in relation to incidence of type 2 diabetes in a Cox proportional hazards model. They concluded that manifest arterial disease patients with high hsCRP plasma levels are at increased risk to develop type 2 diabetes and this risk is more pronounced in female than male patients.

While some epidemiologic studies investigated and found significant interactions or effect modifications, in a majority of epidemiologic studies the interactions or effect modifications are not explored. We estimated the relative frequency of lack of exploring potential interactions in regression analyses by conducting a non-systematic search in PubMed for the period, January 2004 to December 2013. Since interactions and effect modifications are usually explored via multivariable regression models and different terms such as “multivariable regression” or “multiple regressions” or “regression” are used in regression analysis [7], we restricted our search only to “multivariable regression” and “multiple regression”, which are often used interchangeably by epidemiologists. Next, among papers using the terms “multivariable regression”, “multiple regression” or “regression”, we searched for the terms “statistical interaction”, “effect modifier”, “effect modification”, or “heterogeneity of effect”. The result revealed that 4.4% of the published studies in PubMed that used terms “multivariable regression” (or “multiple regression”) have used terms related to interactions, effect modifications, or heterogeneity of effects in their publications.

Although some of the publications may not be related to epidemiologic studies, or the terms interaction or heterogeneity may be used under different context, our search revealed that a majority of investigators may not explore interactions or effect modifications when conducting regression analysis.

The aim of this study was to demonstrate the adverse effects of ignoring interactions in regression models by conducting simulation studies in different scenarios. Specifically, we studied the effect of ignoring interactions in a Cox proportional hazards regression model. In addition, to demonstrate the importance of including interactions in regression models in real life epidemiologic studies, we compared results from additive and interactive models using data from the Cameron County Hispanic Cohort (CCHC) [8]. For this we specifically focused on investigating the role of peripheral white blood cells (WBC) counts, WBC differential counts and BMI in association with the time to incidence of type 2 diabetes while controlling for the effect of other known type 2 diabetes risk factors.

## Materials and Methods

### Simulation study for investigating interactive effects

#### Dataset generation

To assess the impact of ignoring the interactive effects, we simulated data from two fully specified Cox proportional hazards regression models *h*(*t|x*)=*h_0_*(*t*)exp(*β*′*x*) where *h*(*t|x*) is the hazard rate at time t for an individual with risk vector *x*, *β* is the vector of regression coefficients, and *h_0_*(*t*) is the baseline hazard, with two predictor variables that had only additive or interactive effects. In addition different magnitudes for the coefficient of the interaction term were considered. All Cox proportional hazards regression models that were used for simulation are presented in Table 1. For each of four scenarios 1000 replications, with sample sizes of 600 each, were generated in a manner that are described in the following.

In order to simulate data from the underlying Cox models, the effect of the covariates have to be translated from the hazard functions to the survival times, as documented in the literature [9,10]. It can be shown that the survival time for exponential survival random variable could be written as *T=*−In[*S*(*t*)]/[λexp(*β*′*x*)], where S(t) is the probability of surviving beyond time t and λ>0 is the scale parameter of the exponential distribution. When S(T) has a uniform distribution between 0 and 1 (i.e., S(T)~U(0,1)) then −In[*S*(*T*)] is exponentially distributed with parameter 1, and T is exponentially distributed with parameter λ′ (x)=λexp(*β*′*x*). To create survival time data we generated exponentially distributed survival times T with scale parameters λ′ (*x*)=λexp(*β*′*x*) with an arbitrarily chosen constant baseline hazard rate λ=*h_0_* (*t*) [9,10]. Next, we generated exponentially distributed censoring time *c* with arbitrary chosen constant baseline censoring rate λ_1_ [10]. To generate right-censored survival time data the observed time to event was defined as minimum of the survival T and censoring variable C. That is generated observed time to events were (time to event=T when T<C), where the censoring status variable had a value of 1 if the time to event is censored (C>T) or 0 otherwise [11,12]. The parameters of the exponential distribution were determined by several iterations such that the censoring rate in each simulated dataset was higher than 30%. While in Cox proportional hazards regression model the predictor variables are not required to be normally distributed, for this simulation study the pre-specified continuous variable *x_1_* was generated from a normal distribution with mean 6.4 and variance 2.25 that represents the distribution of WBC counts observed in the CCHC data. The pre-specified binary variable *x_2_* was generated as a Bernoulli random variable with probability of success p=0.5, that closely represents the distribution of obese status of individuals in the CCHC subset. The regression coefficients in all scenarios were arbitrarily set to *β_1_=*2 and *β_2_=*1.

#### Model comparisons

Using the simulated datasets Cox models with and without interaction terms were fitted under each of the scenarios. To summarize the simulation results, estimates for each regression coefficient and the corresponding standard errors from the 1000 replications were averaged for each of the scenarios.

For comparing coefficient estimates between correctly specified and misspecified Cox regression models percentage of bias was calculated using $\Delta =\frac{\hat{\beta }-\beta }{\beta }*100$, where $\bar{\hat{\beta }}$ is the average of the estimated regression coefficient β̂ over 1000 replications and *β* is the true value of the regression coefficient. Overall accuracy of the estimates was assessed by mean squared error (MSE) which is the sum of the variance of the estimator and the squared bias (*MSE* = *var*(*β̂*) + (*Bias*(*β̂*,*β*))^2^).

All simulations were performed with Stata 12 [13] using survsim module [14,15] and the statistical analyses were performed using SAS 9.4 [16].

### Empirical example for demonstrating interaction effects using Cameron County Hispanic Cohort Data

To demonstrate the importance of identifying interactions in epidemiologic studies, in this section we referred to an earlier finding from analyses of CCHC data with Cox proportional hazards regression model [8]. Briefly, using data from 636 participants in the CCHC, this study assessed crude and adjusted effects of total and differential WBC counts (lymphocytes, monocytes, and granulocytes), C-reactive protein (CRP), and Body Mass Index (BMI) in relation to time to progression to type 2 diabetes in Mexican Americans. All participants without any evidence of type 2 diabetes after the baseline visit, death or lost-to-follow up were considered as censored at their last observation dates. Other covariates, such as age at the time of the event or censoring, gender, family history of diabetes, pre-diabetes status, smoking, and triglycerides were also included in the regression analysis. The effect modification of continuous BMI and BMI levels (e.g., BMI<25 (normal), 25 ≤ BMI<30 (overweight), and BMI ≥30 (obese)) on total and differential WBC counts were evaluated. In this study, the presence of the interactions was tested by including the product of the dichotomized BMI as BMI ≥35 and BMI <35 with total or differential WBC counts in the models [17] and by using a likelihood ratio test for the nested models with and without an interaction term. Also, we conducted multivariable Cox proportional hazards regression models stratified analyses by BMI categories to compare stratum specific hazard ratios with those obtained from our final interactive Cox proportional hazards regression models.

Cox proportional hazards regression model assumptions for the functional form of the continuous covariates (e.g., the linear relationship between log cumulative hazard and a covariate) and proportionality assumption of the hazards of all covariates were evaluated before and after including the interaction term in the model. Our result indicated that family diabetes history and pre-diabetes status did not satisfy the proportional hazards assumption; hence all models that included these two variables were stratified by these variables by including strata statement in SAS Proc Phreg [18]. Details regarding the model development are described elsewhere [8].

### Calculation of hazard ratios in the presence of interactive effects

The calculations of Hazard Ratios (HRs) from an interactive Cox proportional hazards model is different from the calculations of HRs from an additive model. For example in the following interactive Cox proportional hazards model.

(1) $$h\;(t|x)=h_{0}\;(t)\exp\;(\beta_{1}x_{1}+\beta_{2}x_{2}+\beta_{3}x_{1}x_{2}),$$

Where x_1_ is a continuous variable and x_2_ is a dichotomous variable with values 1 or 0, the HRs for x_1_ by the levels of variable x_2_ in (1) can be written as HR=exp (*x_1_* (β_1_ + 0β_3_))=exp (*x_1_*β_1_) when x_2_=0, and the HR for *x_1_* is HR=exp (*x_1_* (β_1_ + β_3_)) when x_2_=1.

In contrast in an additive Cox proportional hazards regression model.

(2) $$h\;(t|x)=h_{0}\;(t)\exp\;(\beta_{1}x_{1}+\beta_{2}x_{2})$$

the HR for *x_1_* is exp (*x_1_*β_1_) adjusted for the effect of the covariate *x_2_*.

## Results

### Simulation study

Table 2 shows the results of the analysis of simulated data with Cox proportional hazards regression model (1) without the interaction term. The coefficient estimate for variable *x_1_* in the correctly specified (model with no interaction term) and misspecified model (model with interaction term) had the same percentage of bias (0.5%) and MSE (0.01). However the estimate for coefficient of variable *x_2_* in the misspecified model was more biased (2%) and less accurate (MSE=0.72) compared to the correctly specified model (bias=0%, MSE=0.02). As expected the estimate for coefficient of the interaction term in the misspecified regression model (i.e., true model was additive but we fitted an interactive model) was very small and negligible which led to very similar estimates of 2.013 for the true coefficient of variable *x_1_* that was 2. The estimated coefficient of variable *x_1_* was 2.01 when adjusted for the effect of variable *x_2_* in the correctly specified model. As expected the model with interaction term did not impact *x_1_* or *x_2_* estimates significantly. In addition based on the interactive (misspecified) model when *x_2_=*0 the HR for *x_1_* is 7.49 and when *x_2_*=1 the HR for *x_1_* is 7.46. Whereas based on the additive (correct) model the HR for *x_1_* is 7.46, adjusted for the effect of *x_2_*. This illustrates that in the case of a perfect additive Cox proportional hazards regression model the inclusion of the interaction of the covariates does not have a significant impact on main effect regression coefficients estimates, hence on the estimated hazard ratios.

Table 3 shows the results of the analyses on simulated data with Cox proportional hazards models with interaction term under scenarios (2), (3) and (4). Compared to the correctly specified models, the regression coefficient estimates for variables *x_1_* and *x_2_* in the misspecified models in all scenarios were more biased and less accurate as the magnitude of the coefficient of the interaction term increased [10]. Specifically, the bias increased from 352% to 894% and MSE increased from 12.43 to 80.18, as the magnitude of the coefficient of the interaction term increased from 0.5 to 1.5.

### Findings from the empirical study

The results from the analyses of CCHC data are presented in Tables 4 and 5 including estimates for the coefficients and their standard errors. In our final models we identified significant interactions between dichotomous BMI (i.e., obese categories) and total WBC (p-value=0.0137), granulocytes (p-value=0.0291), and lymphocytes (p-value=0.0478) after adjusting for the effect of age at the time of the event or censoring, gender, smoking, and triglycerides and stratified for family history of diabetes and pre-diabetes status by including strata statement in SAS Proc Phreg. Because of the significant interaction between dichotomous BMI and total WBC, counts, granulocytes, and lymphocytes, we calculated estimated adjusted hazard ratios for WBC counts, granulocytes, and lymphocytes by different levels of the interacting variable BMI.

**Figure 1:**
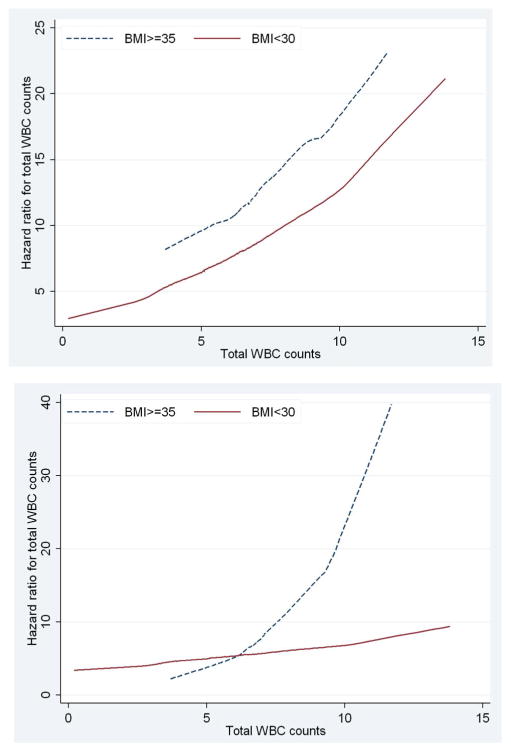
Estimated Adjusted Hazard Ratios for total WBC counts in relation to time to progression to type 2 diabetes in Mexican Americans by the levels of BMI. (a) Excluding the interaction term between BMI and WBC. (b) Including the interaction term between BMI and WBC.

**Figure 2:**
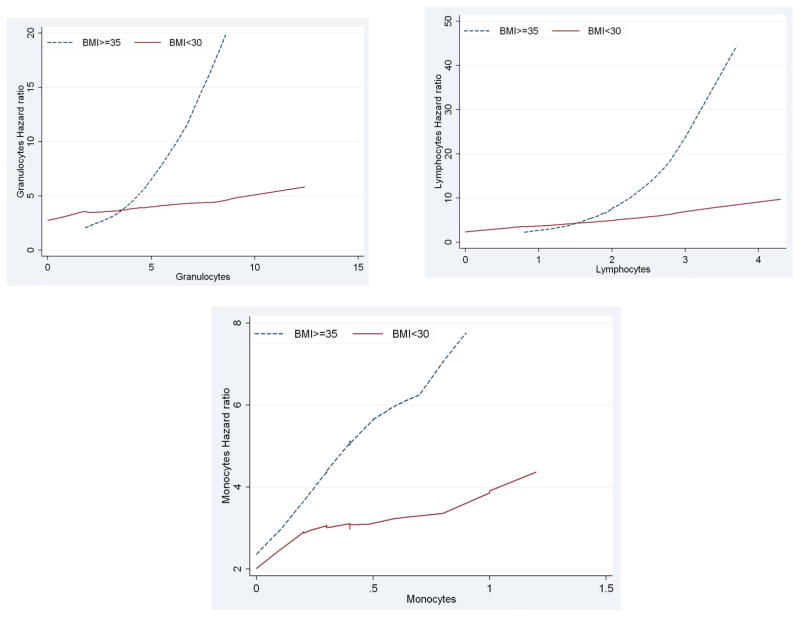
Estimated Adjusted Hazard Ratios in relation to time to progression to type 2 diabetes by the levels of BMI for (a) Granulocytes, (b) Lymphocytes and (c) Monocytes.

Figure 1a and 1b present the plots of estimated adjusted hazard ratios for total WBC counts for BMI ≥35 vs. BMI <35 under a model with no interaction and a model including interaction term between BMI and total WBC counts, Under the additive model (i.e., with no interactions) the lines for the hazard ratios were somewhat parallel and the vertical distance between them represented the BMI adjusted hazard ratio for WBC counts (Figure 1a). The plot based on the interactive model (Figure 1b) shows departure from being parallel (i.e., constant adjusted Hazard Ratio) which was confirmed with the significant interaction between BMI levels and WBC (p-value=0.0137). The fact that the line for BMI ≥35 lies above the line for BMI<35 showed that the adjusted HR for BMI ≥35 was greater than the adjusted HR for BMI<35 and this was confirmed for total WBC counts of greater than 5. The estimated adjusted HR for WBC in the model with no interaction after adjusting for the effect of BMI and the other diabetes risk factors was 1.09 [95% CI (0.97, 1.24)]. In the interactive model WBC was associated with progression to diabetes in participants with BMI ≥35 and the estimated adjusted HR was 1.49 [95%CI (1.20, 1.85)] while WBC was not significantly associated with progression to diabetes in participants with BMI <35 and the estimated adjusted HR was 1.09 [95%CI (0.97, 1.85)]. Similarly, Figure 2a and 2b with two non-parallel lines present the HRs of granulocytes and lymphocytes respectively by the levels of BMI under the models with interaction, adjusting for the effect of age at the time of the event or censoring, gender, smoking, and triglycerides and stratified by family history of diabetes and pre-diabetes status by including strata statement in SAS Proc Phreg. Finally, Figure 2c presents the interaction effect between monocytes and BMI in relation to time to progression to type 2 diabetes and it shows that when monocytes were between 0 and 0.4, and 0.8 and 1.1 the HR lines for BMI ≥35 and BMI<35 are approximately parallel, and the interaction effect between the two variables was not statistically significant (p-value=0.16).

The results from stratified analyses with multivariable Cox proportional hazards regression models by BMI categories are reported in Table 5 which, are similar to those of the adjusted interactive models in Table 4. The results in Table 5 reconfirm that the significant associations of total WBC counts, lymphocytes and granulocytes with progression to type 2 diabetes were only among participants with BMI ≥35. Furthermore, the stratum specific HR estimates for each of the variables in Table 5 were not similar indicating the presence of interaction effects of BMI with total WBC counts, lymphocytes and granulocytes.

## Discussion

In this paper we have demonstrated that a majority of epidemiologist and scientists do not report whether they have explored potential interactive effects when reporting their findings in publications that involve regression analysis. We have also examined the adverse effects of ignoring interactions when using a Cox proportional hazards regression model. We used simulation studies and data from the Cameron County Hispanic Cohort, to demonstrate detection, calculation of effects based on interactive models, and graphical presentation of interactive effects. The approach for the simulation studies was to first generate data from an additive model with known distributions and apply an additive (without their product term) Cox proportional hazards regression model with two covariates *x_1_* and *x_2_* to estimate the regression parameters. In this scenario the relationship between the variable *x_1_* and the time to event did not depend on the values of variable *x_2_*. Therefore in the perfect additive Cox proportional hazards regression model the inclusion of the interaction of the two covariates did not have a significant impact on main effects of regression coefficients estimates, hence on the estimated hazard ratios and their interpretation. In the second scenario data were generated using Cox proportional hazards regression model with two covariates *x_1_* and *x_2_* and their product term (Model 2). The analyses demonstrated that the misspecified models with no interaction term resulted with biased regression coefficient estimates, hence erroneous interpretations. In the interactive model the effect of *x_1_* on time to event was dependent on the levels of variable *x_2_* and we demonstrated how to calculate the adjusted HRs in the presence of interactions between *x_1_* and *x_2_*.

Since this study focused on recognition of statistical interaction in regression analysis, in this paper we do not fully discus the findings from the CCHC data. However, the results from the CCHC prospective cohort study demonstrated with real life variables the difference between models with and without interaction term, more specifically the improvement of the inferences for hazard ratios for total WBC counts, granulocytes and lymphocytes after testing and identifying their interaction with dichotomous BMI (BMI ≥35 vs. BMI <35). The models with interaction terms revealed that WBC counts, lymphocytes and granulocytes were significant risk factors in the subjects with BMI ≥35. The presence of the interaction effects and the positive statistical association between total WBC counts, granulocytes and lymphocytes and progression to diabetes in individuals with BMI ≥35 were confirmed when Cox proportional hazards regression models fitted separately in the BMI strata. Stratification is not only a technique to evaluate the presence of possible confounding but also allows for an evaluation of possible presence of interaction effect [1,2].

A key underlying assumption (assumption of proportional hazards) of the Cox proportional hazards model is that the hazard ratio is constant over time [1,18]. In our simulation studies and in the CCHC study the interactive variables met the proportional hazards assumption. However, stratification by the variable where this assumption does not hold is a popular solution to the problem by including strata statement in SAS Proc Phreg.

### Strengths and Limitations

With the analyses of the simulated datasets and CCHC data we highlighted that failure to test for interaction effects in a regression analysis does lead to a significant bias in parameter estimates due to unaccounted interaction effect when it actually exists. We have also demonstrated calculation and interpretation of effects based on parameters estimates in a Cox regression models with a significant interactive effect. Statistical guidelines for best practices recommend assessment of interactions as one of the steps during the model building process [17,19]. Although the work presented here is based on Cox regression models, the findings from this study are generalizable to other regression models including linear, logistic, Poisson, and negative binomial regression models.

Our studies have some limitations. These studies illustrated a two-way interaction effect between a continuous and a dichotomous variable. In practice interaction effect may occur between continuous or categorical variables and it may even be represented by a higher order product terms. In addition in the Cox regression models to handle non-proportional hazards we used stratification in the variable where non-proportional hazards assumption was not met. Another method to handle non-proportional hazards is adding an interaction term between some function of time variable and the covariate which takes into consideration the non-constant influence of covariate on the hazard [20].

Since the main purpose of the study was to demonstrate the adverse effects of ignoring statistical interactions in regression models used in epidemiologic studies we did not use the developed sampling weights from the CCHC which were created to account for imbalances in the distribution of sex and age due to unequal participation of household members in the census tracts and to scale the sample to the population [21].

## Conclusions

Our findings from a non-systematic review for this study revealed that a majority of published literature on epidemiologic studies that used multivariable regression models have not mentioned anything related to testing for statistical interactions, effect modification, or heterogeneity of effect. Although calculation and interpretation of interactive effects are more difficult these are essential if the effects are interactive or synergic. We recommend inclusion of interaction terms that are clinically significant even if the interaction effects are not statistically significant. The failure to identify interactive effects in regression models could lead to significant bias, misinterpretation of the results, and in some instances to incorrect public health interventions with potential adverse implications.

## Figures and Tables

**Table 1:** Cox proportional hazards regression models used for different scenarios of the simulation study.

Scenario	Cox proportional hazards regression model*
1	h(t|x)=h_0_(t) exp (2x_1_+x_2_)
2	h(t|x)=h_0_(t) exp (2x_1_+x_2_+0.5x_1_x_2_)
3	h(t|x)=h_0_(t) exp (2x_1_+x_2_+x_1_x_2_)
4	h(t|x)=h_0_(t) exp (2x_1_+x_2_+1.5x_1_x_2_)

* The values of the regression coefficients were arbitrarily set for simulations.

**Table 2:** Findings from multivariable Cox proportional hazards regression model fitted using generated data in scenario 1.

Parameter estimates	Correct Model†	Misspecified Model‡
Estimated coefficient of X_1_ ± SE	2.01 ± 0.09	2.01 ± 0.11
%bias in estimated coefficient of X_1_	0.50%	0.50%
MSE*	0.01	0.01
Estimated coefficient of X_2_ ± SE	1 ± 0.12	0.98 ± 0.85
%bias in estimated coefficient	0.00%	−2.00%
MSE*	0.02	0.72
Estimated coefficient of X_1_* X_2_ (1 vs. 0) ± SE	-	0.003 ± 0.11

† Correct model is model with *no interaction term* X_1_*X_2_ included;

‡ Misspecified model is the model that *include interaction* term X_1_*X_2_ in addition to X_1_ and X_2_ main effects;

* Mean squared error is defined as the sum of the variance of the estimator and the squared bias.

**Table 3:** Findings from multivariable Cox proportional hazards regression models fitted using generated data in scenarios 2, 3 and 4.

Parameter estimates	Data generated with b_3_=0.5 (Model 2)		Data generated with b_3_=1.0 (Model 3)		Data generated with b_3_=1.5 (Model 4)	
	Misspecified Model†	Correct Model‡	Misspecified Model†	Correct Model‡	Misspecified Model†	Correct Model‡
Estimated coefficient of X_1_ ± SE	2.26 ± 0.09	2.01 ± 0.11	2.42 ± 0.09	2.01 ± 0.11	2.52 ± 0.1	2.01 ± 0.12
%bias in estimated coefficient	13.00%	0.50%	21.00%	0.50%	26.00%	0.50%
MSE*	0.08	0.01	0.19	0.01	0.29	0.01
Estimated coefficient of X_2_ ± SE	4.52 ± 0.21	1 ± 0.83	7.51 ± 0.3	1.04 ± 0.91	9.94 ± 0.39	1.03 ± 1.01
%bias in estimated coefficient	352.00%	0.00%	651.00%	4.00%	894.00%	3.00%
MSE*	12.43	0.7	42.49	0.85	80.18	1.03
Estimated coefficient of X_1_* X_2_ ± SE	-	0.5 ± 0.12	-	1 ± 0.14	-	1.5 ± 0.16
%bias in estimated coefficient		0.00%		0.00%		0.00%
MSE*		0.01		0.02		0.03

† Misspecified model is the model with no interaction term X_1_*X_2_ included

‡ Correct model is the model with interaction term X_1_*X_2_ as well as the main effects of X_1_ and X_2_

* Mean squared error is defined as the sum of the variance of the estimator and the squared bias

**Table 4:** Findings (SI units) from interactive multivariable Cox proportional hazards regression models of time to first type 2 diabetes using Cameron County Hispanic Cohort data, 2003–2014.

Variables	Unadjusted models*	Adjusted models*
	HR (95% CI)	HR (95% CI)
Models with total WBC counts ×10^9^/L: Hazard ratio for WBC by the levels of the interacting variable BMI		
WBC in BMI≥35	1.29 (1.07–1.55)	1.49 (1.20–1.85)
WBC in BMI<35	1.09 (0.97–1.23)	1.09 (0.97–1.85)
Models with Lymphocytes ×10^9^/L: Hazard ratio for Lymphocytes by the levels of the interacting variable BMI		
Lymphocytes in BMI≥35	2.60 (1.49–4.53)	2.95 (1.57–5.52)
Lymphocytes in BMI<35	1.45 (0.98–2.12)	1.43 (0.98–2.09)
Models with Monocytes ×10^9^/L: Hazard ratio for Monocytes by the levels of the interacting variable BMI		
Monocytes in BMI≥35	3.36 (0.46–24.64)	3.71 (0.44–31.19)
Monocytes in BMI<35	1.88 (0.41–8.68)	1.73 (0.40–7.42)
Models with Granulocytes ×10^9^/L: Hazard ratio for Granulocytes by the levels of the interacting variable BMI		
Granulocytes in BMI≥35	1.26 (1.00–1.60)	1.49 (1.14–1.94)
Granulocytes in BMI<35	1.06 (0.92–1.22)	1.07 (0.92–1.23)
Models with Granulocytes ×10^9^/L and Lymphocytes ×10^9^/L: Hazard ratio for Granulocytes by the levels of the interacting variable BMI		
Granulocytes in BMI≥35	1.2 (0.95–1.52)	1.46 (1.19–1.92)
Granulocytes in BMI<35	1.02 (0.88–1.20)	1.02 (0.86–1.20)

* Models adjusted for age, gender, smoking, and triglycerides and stratified by family history of diabetes and pre-diabetes status by including strata statement in SAS Proc Phreg.

**Table 5:** Findings (SI units) from multivariable Cox proportional hazards regression models* for time to first type 2 diabetes among participants in BMI stratified Cameron County Hispanic Cohort, 2003–2014.

Variables	BMI groups	
	BMI<35	BMI ≥35
	HR (95% CI)	HR (95% CI)
Models with total WBC counts ×10^9^/L		
WBC	1.10 (0.97, 1.24)	1.47 (1.14, 1.89)
Models with Lymphocytes ×10^9^/L		
Lymphocytes	1.43 (0.98, 2.1)	3.25 (1.58, 6.7)
Models with Monocytes ×10^9^/L		
Monocytes	1.80 (0.43, 7.55)	3.13 (0.38, 26)
Models with Granulocytes ×10^9^/L		
Granulocytes	1.07 (0.92, 1.24)	1.42 (1.06, 1.9)
Models with Granulocytes ×10^9^/L and Lymphocytes ×10^9^/L		
Granulocytes	1.04 (0.89, 1.22)	1.36 (1.002, 1.83)

* Models adjusted for age, gender, smoking, and triglycerides and *stratified* by family history of diabetes and pre-diabetes status by including strata statement in SAS Proc Phreg.
